# Plasma and follicular fluid osteopontin levels during ovarian cycle and their correlation with follicular fluid vascular endothelial growth factor levels

**DOI:** 10.1038/s41598-020-79453-1

**Published:** 2021-01-11

**Authors:** Yoshimitsu Kuwabara, Shuichi Ono, Akira Katayama, Sachiko Kurihara, Yumiko Oishi, Toshiyuki Takeshita

**Affiliations:** 1 Department of Obstetrics and Gynecology, Nippon Medical School, 1-1-5 Sendagi, Bunkyo-ku, Tokyo 113-8602 Japan; 2 Department of Biochemistry and Molecular Biology, Nippon Medical School, 1-1-5 Sendagi, Bunkyo-ku, Tokyo 113-8602 Japan

**Keywords:** Physiology, Biomarkers, Endocrinology, Medical research

## Abstract

Osteopontin (OPN) is a multifunctional secreted glycoprotein. We evaluated OPN concentrations in blood and follicular fluid (FF) during the ovarian cycle and their relationship with the production of vascular endothelial growth factor (VEGF), which is involved in the pathophysiology of ovarian hyperstimulation syndrome (OHSS). Twenty-two women undergoing in vitro fertilization (minimal stimulation protocol with clomiphene citrate) were enrolled. Samples were collected (a) on the third day of withdrawal bleeding, (b) 2 days before oocyte retrieval, and (c) on the day of oocyte retrieval. FF was collected during oocyte retrieval. The OPN concentration in each specimen and the VEGF concentration in FF was measured by enzyme-linked immunosorbent assays. Plasma OPN concentrations were (in ng/mL): (a) 416 ± 37.2, (b) 378 ± 35.8, and (c) 390 ± 40.0, with no significant differences between the groups. The OPN concentration in FF was 106 ± 13.4 ng/mL. A positive correlation was found between OPN concentrations in FF and plasma samples. A positive correlation was also found between plasma OPN and FF VEGF concentrations, irrespective of the blood-sampling period. Plasma OPN concentration is suggested to reflect the FF VEGF level at oocyte retrieval and maybe a novel clinical marker for predicting the risk for OHSS.

## Introduction

Osteopontin (OPN) is a highly phosphorylated secreted multifunctional glycoprotein first identified as an extracellular matrix protein in bone tissue^1^. OPN is reportedly distributed in a variety of tissues^2–4^ and is involved in various physiological processes including inflammation, biomineralization, cell viability, and wound healing^5^. Previously, we reported that the expression of *Opn* was markedly upregulated in the mouse ovarian granulosa cell layer in response to a gonadotropin surge through epidermal growth factor receptor signaling. We further provided experimental evidence showing that OPN promoted progesterone synthesis and vascular endothelial growth factor (VEGF) production through phosphoinositide 3-kinase/AKT signaling during the early luteal phase^6^. Elucidating the mechanism of regulation of ovarian VEGF is an important issue in reproductive medicine because VEGF is an essential molecule involved in follicular development and luteinization, and in the pathophysiology of ovarian hyperstimulation syndrome (OHSS) characterized by increased vascular permeability^7^. OHSS is a severe health complication observed in patients undergoing in vitro fertilization (IVF). Although a higher number of follicles are indicators associated with this syndrome, they may not be sufficient to predict the onset and severity. Therefore, we hypothesized that OPN produced by follicular tissue may promote VEGF production through autocrine actions during early luteinization in humans and may also be involved in the pathophysiology of OHSS.

To date, there have been few studies reporting blood OPN levels during the human ovarian cycle, or the presence and concentration of OPN in human follicular fluid. In this study, we evaluated plasma OPN levels from the follicular to periovulatory periods during IVF with clomiphene citrate (CC). We also measured OPN and VEGF levels in follicular fluid collected during oocyte retrieval. We aimed to evaluate the role of OPN in promoting the production of VEGF in humans, as indicated in our previous experiment on mice.

## Materials and methods

### Subjects and study design

This investigation was conducted according to the principles stated in the Declaration of Helsinki*.* The study protocols were approved by the Medical Ethics Committee of Nippon Medical School Hospital. Twenty-two women undergoing IVF with a minimal stimulation protocol with CC participated in this study after providing informed consent using a paper-based written form.

CC (50 mg/day) was administered orally as an extended regimen from cycle day 3 until the induction of oocyte maturation. Follicle growth was monitored by ultrasonography and the serum levels of hormones, including estradiol (E_2_), progesterone, follicle stimulating hormone (FSH), and luteinizing hormone (LH), were measured. Maturation was triggered by administration of 600 μg of a gonadotropin releasing hormone agonist (buserelin) nasal spray when the diameter of the dominant follicle reached or exceeded 18 mm. Ultrasound-guided transvaginal egg retrieval was performed 32–36 h later. Sample collection was performed at the following time points: (a) on the third day of withdrawal bleeding, (b) 2 days before oocyte retrieval, and (c) on the day of oocyte retrieval. Blood was collected in Spitz tubes containing EDTA and centrifuged at 2500 rpm for 15 min. The supernatant was transferred to a separate container and stored at − 70 °C until the measurement. Follicular fluid collected during oocyte retrieval was processed in the same way.

### Measurements of OPN and VEGF levels

Plasma OPN levels were measured (a) on the third day of withdrawal bleeding, (b) 2 days before oocyte retrieval, and (c) on the day of oocyte retrieval. Follicular fluid was collected during oocyte retrieval. OPN levels in human plasma and follicular fluid were assayed with a human OPN enzyme-linked immunosorbent assay (ELISA) kit (IBL, Minneapolis, MN, USA; Cat# 27158) according to the manufacturer’s instructions. VEGF levels in follicular fluid were determined with a VEGF ELISA kit (IBL; Cat# 27171) according to the manufacturer’s instructions.

### Statistical analyses

Relationships between parameters were tested using the Pearson product-moment correlation coefficient. Group medians were compared using the Mann–Whitney *U*-test for independent data. Results were considered statistically significant at p < 0.05. Data are expressed as means ± SE.

## Results

### Characteristics of the study population

Characteristics of the women participants in this study are shown in Table 1. Blood sampling tests for FSH, LH, and E_2_ were performed during the third day of the menstrual cycle, and for AMH and PRL were performed regardless of the menstrual cycle. Four patients had endometriosis and the remaining women had unexplained infertility. IVF was recommended because pregnancy failed to establish after several instances of timed intercourse and/or intrauterine insemination. All women participating in this study had a normal menstrual cycle without polycystic ovary syndrome. There were no cases of endocrine abnormalities, abnormal glucose tolerance, or thyroid dysfunction.

**Table 1:** Demographic and laboratory characteristics of the subjects.

Variables	n = 22 (means ± SE)
Age	36.10 ± 4.91
BMI	22.28 ± 0.54
AMH	3.35 ± 2.76
FSH (mIU/mL)	10.4 ± 3.54
LH (mIU/mL)	4.15 ± 1.86
E_2_ (pg/mL)	25.96 ± 16.34
PRL (ng/mL)	13.64 ± 3.77

BMI, body mass index; AMH, anti-Müllerian hormone; FSH, follicular stimulating hormone; LH, luteinizing hormone; E_2_, estradiol; PRL, prolactin.

### Comparison of plasma OPN levels in the menstrual, late follicular, and periovulatory phases

**Figure 1:**
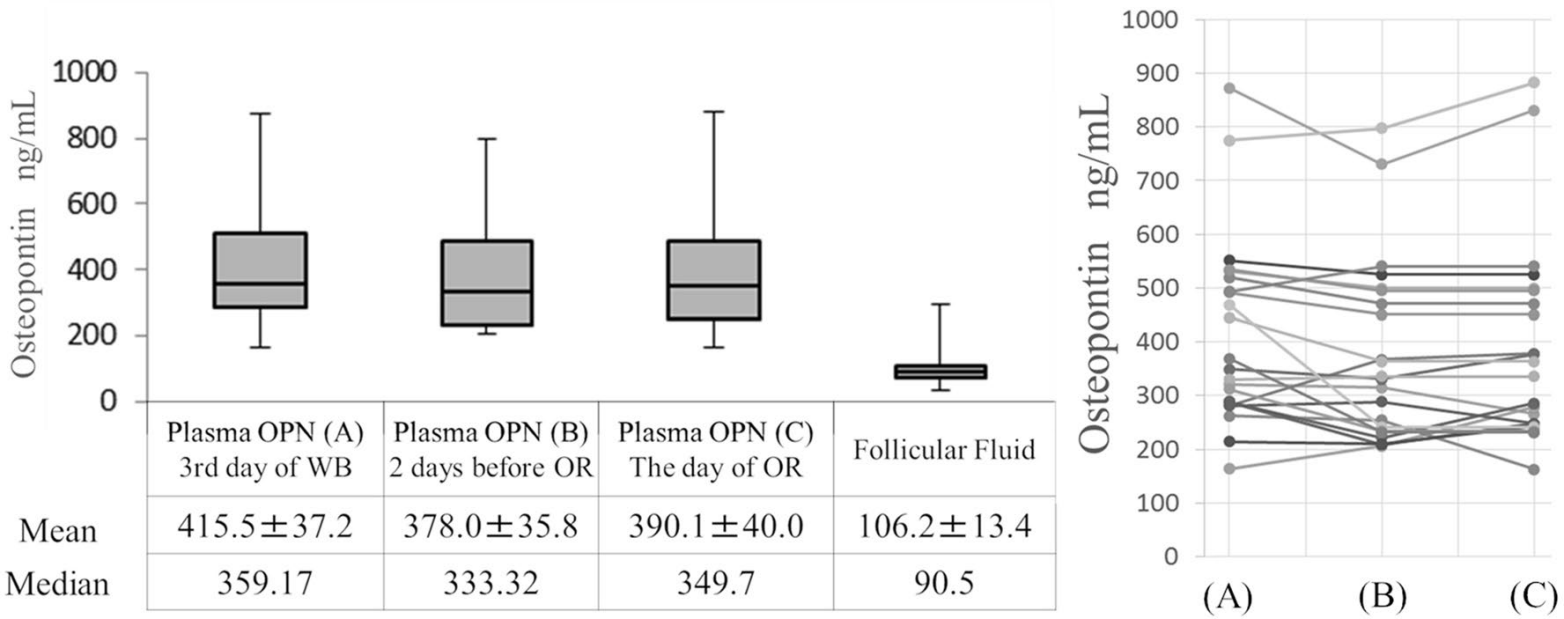
OPN concentrations in plasma and follicular fluid during the clomiphene citrate-stimulated IVF cycle. The results are shown as a box plot graph (left-hand side) and a line chart showing changes in hormone levels in individual cases (right-hand side). The blood OPN concentration did not vary during the follicular to periovulatory phases (p < 0.05). A fourfold gradient is evident between the concentration of OPN in blood and that in follicular fluid. Values shown are means ± SE. OPN, osteopontin; IVF, in vitro fertilization; FF, follicular fluid; SE, standard error, WB, withdrawal bleeding; OR, oocyte retrieval.

**Figure 2:**
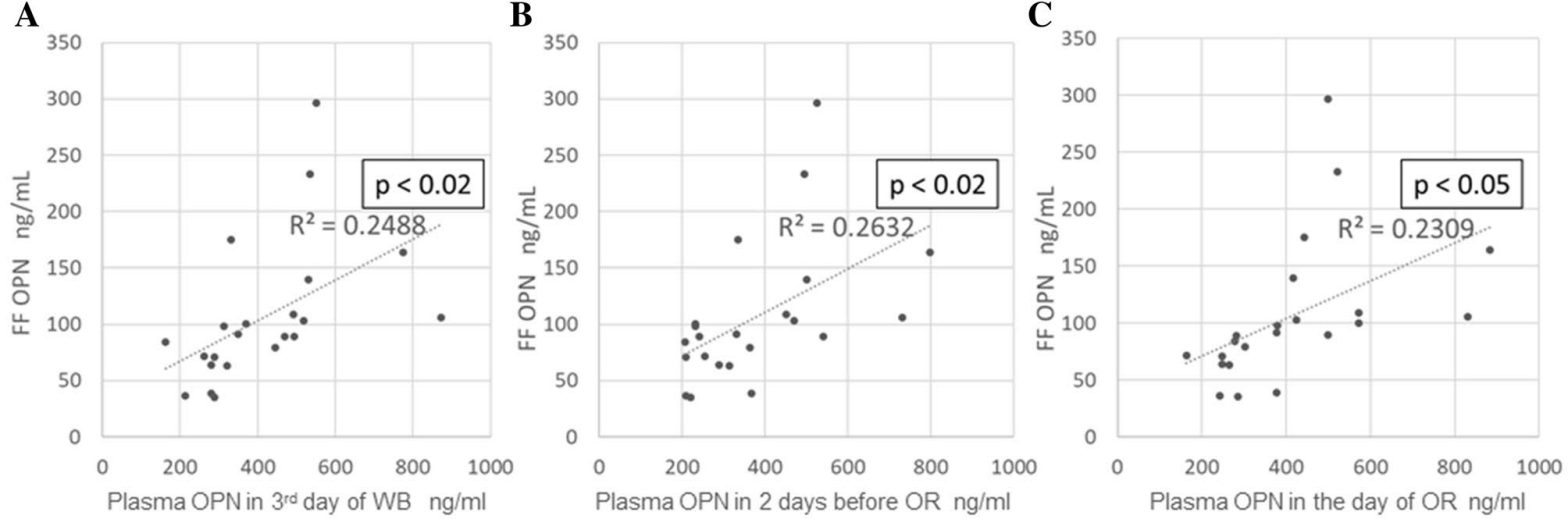
Correlation between OPN concentrations in plasma and follicular fluid. Plasma OPN concentrations were measured at the following time points: (**A**) on the third day of withdrawal bleeding, (**B**) 2 days before oocyte retrieval, and (**C**) on the day of oocyte retrieval. A positive correlation was observed between OPN concentrations in plasma samples and those in follicular fluid samples irrespective of the blood-sampling period. OPN, osteopontin; FF, follicular fluid, WB, withdrawal bleeding; OR, oocyte retrieval.

OPN levels were measured for all patients during the menstrual, late follicular, and periovulatory phases. Plasma OPN concentrations (all in ng/mL) were as follows: (a) on the third day of withdrawal bleeding: 416 ± 37.2 (median: 359), (b) 2 days before oocyte retrieval: 378 ± 35.8 (median: 333), and (c) on the day of oocyte retrieval: 390.1 ± 40.0 (median: 350). No significant differences were found in OPN levels between these groups (Fig. 1). The OPN concentration in follicular fluid was 106 ± 13.4 ng/mL (median: 90.5 ng/mL). Positive correlations of OPN concentrations were found between each plasma sample and follicular fluid samples (a: r = 0.48, p < 0.01; b: r = 0.51, p < 0.02; c: r = 0.49, p < 0.05) (Fig. 2).

### Correlation analyses of follicular fluid VEGF vs. follicular fluid OPN and follicular fluid VEGF vs. plasma OPN concentrations

**Figure 3:**
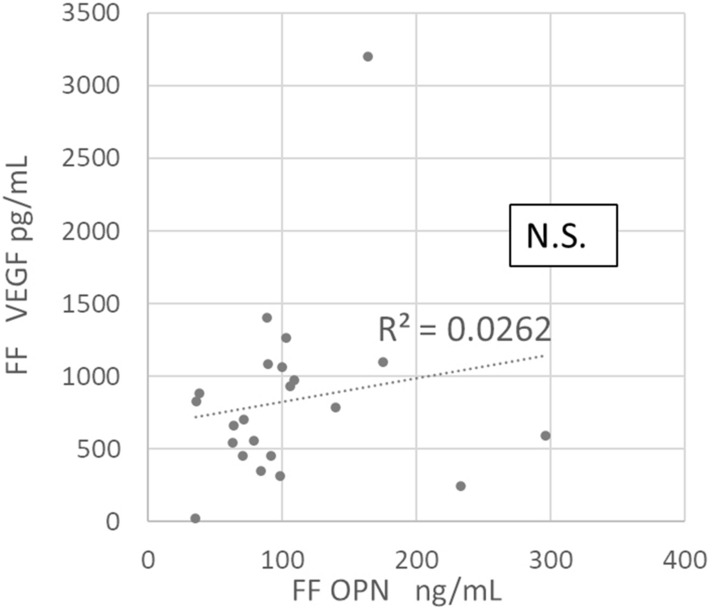
Correlation between molecular concentrations of VEGF and OPN in follicular fluid. The VEGF concentration in follicular fluid does not correlate with the OPN concentration in follicular fluid. OPN, osteopontin; VEGF, vascular endothelial growth factor; N.S., non-significant (p > 0.05).

**Figure 4:**
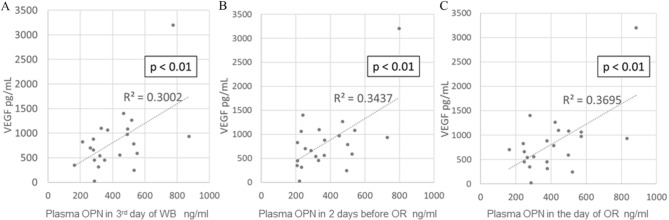
Correlation between the concentrations of follicular fluid VEGF and plasma OPN. Plasma OPN concentrations were measured at the following time points: (**A**) on the third day of withdrawal bleeding, (**B**) 2 days before oocyte retrieval, and (**C**) on the day of oocyte retrieval. The VEGF concentration in follicular fluid positively correlates with the OPN concentration in the plasma irrespective of the blood-sampling period. OPN, osteopontin; FF, follicular fluid, WB, withdrawal bleeding; OR, oocyte retrieval. VEGF, vascular endothelial growth factor.

The follicular fluid VEGF concentration did not correlate with the follicular fluid OPN concentration (Fig. 3); however, it correlated positively with the plasma OPN concentration irrespective of the timing of blood sampling (a: r = 0.65, p < 0.01; b: r = 0.79, p < 0.01; c: r = 0.77, p < 0.01) (Fig. 4).

### Correlation analyses of follicular fluid VEGF vs. serum parameters related to OHSS risk

**Figure 5:**
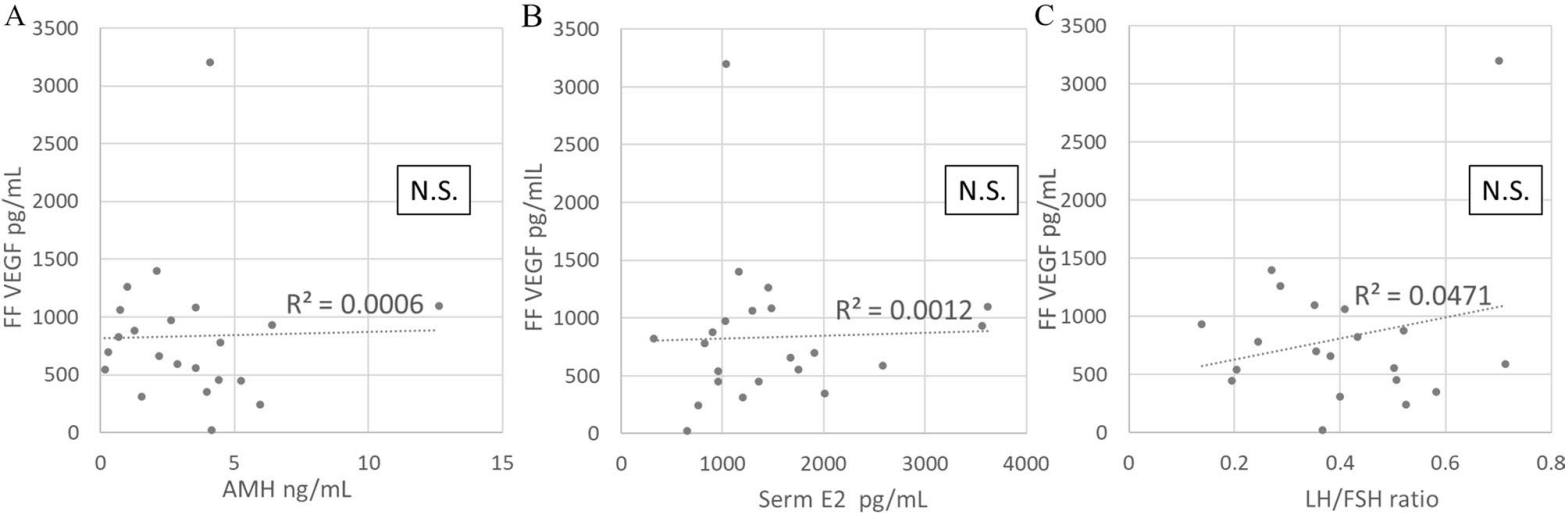
Correlation analyses of follicular fluid VEGF vs. serum parameters related to the risk of OHSS. No correlation was found between the VEGF concentration in follicular fluid and serum predictive factors of OHSS. (**A**) AMH. (**B**) Estradiol 2 days before oocyte retrieval. (**C**) LH/FSH ratio on the third day of withdrawal bleeding. AMH, anti-Müllerian hormone; FSH, follicular stimulating hormone; LH, luteinizing hormone; E_2_, estradiol; FF, follicular fluid; OHSS, ovarian hyperstimulation syndrome; VEGF, vascular endothelial growth factor; N.S., non-significant (p > 0.05).

No correlation was found between the VEGF concentration in follicular fluid and serum predictive factors of OHSS, including anti-Müllerian hormone (AMH), estradiol 2 days before oocyte retrieval, and the LH/FSH ratio on the third day of withdrawal bleeding (Fig. 5).

## Discussion

OPN is produced by various bodily tissues and constantly circulates in the blood. Previously, we reported that remarkable OPN expression in the periovulatory ovary was accompanied by a peak in OPN blood levels in a mouse hyperovulation model^6^. In the current study, we targeted the IVF cycle with mild ovarian stimulation using clomiphene citrate because it is considered closer to the physiological cycle as compared to hyperstimulation with gonadotropin preparations. We found that the blood OPN concentration was constant during follicular to periovulatory phases. This result conflicts with that of a previous study on 40 women with normal menstrual cycles, wherein serum OPN levels were significantly higher during the mid-cycle (Days 13–15) than during the menstrual period^8^. However, such an evaluation using serum is inappropriate as OPN has a conserved thrombin cleavage site at Arg168/Ser169 adjacent to the RGD domain and is potentially cleaved during blood coagulation^9^. Indeed, the mean OPN value reported in serum was 40 –45 ng/mL during the ovarian cycle^8^, which is several times lower than that measured in plasma in the current study. The results of our study suggested that the blood OPN level does not change during the physiological ovarian cycle. Therefore, the effect of OPN produced from the ovary during the periovulatory period on plasma OPN concentration is considered small and it is reasonable to presume that the main source of plasma OPN is a variety of systemic tissues secreting this molecule. Examining a change in blood OPN levels during the ovarian cycle with hyperstimulation using gonadotropin preparations, which is similar to mouse experiments, is an issue to be addressed in the future.

To our knowledge, this is the first study to report the concentration of OPN in follicular fluid. The protein profile in follicular fluid includes proteins produced and transferred from follicular tissue as well as those circulating in the blood that enter the follicular antrum by selective permeability of the blood-follicular barrier (BFB)^10^. The OPN concentration in follicular fluid correlated with that in plasma regardless of the timing of blood sampling, suggesting that this concentration reflects that of OPN in blood. Some amount of OPN produced from ovarian granulosa cells in response to surge of LH may be transferred into the follicular fluid. However, OPN concentration in follicular fluid will eventually be determined according to the concentration gradient of BFB, and is thus dependent on the blood OPN concentration. As plasma OPN concentration is constant regardless of the ovarian cycle, all three points of plasma OPN show a positive correlation with the OPN concentration in follicular fluid collected during the periovulatory period.

Interestingly, there exists about a fourfold gradient between the OPN concentration in blood and follicular fluid despite of its relatively low molecular weight (44–75 kDa, consisting of 314 amino acids)^5^. This can be explained by the presence of rich sugar chain modifications in the protein, which may inhibit its passage through the BFB. As an extracellular matrix protein, OPN is likely to distribute to the cellular interstitium, and this might also contribute to the poor transfer of OPN derived from follicular tissue. On the other hand, plasma VEGF levels in the blood circulation increase in response to the LH surge^11^, and the VEGF concentration in follicular fluid is several times higher than that in serum during the ovulatory phase^12^. Therefore, in contrast to OPN, the VEGF concentration in circulating blood and follicular fluid during this period may reflect that produced by follicular tissue.

In our previous study, OPN enhanced the production of VEGF in primary cultured mouse granulosa cells stimulated with human chorionic gonadotropin^6^. Therefore, to test our hypothesis in humans, we measured the OPN and VEGF levels in follicular fluid and plasma of patients undergoing IVF. However, in this study, there was no significant correlation observed between the OPN and VEGF concentrations in follicular fluid. This could be explained, to some extent, by the above-described theory that the OPN concentration in follicular fluid does not directly reflect that produced locally by follicular tissue. However, serum OPN levels correlated significantly with VEGF levels in follicular fluid obtained during oocyte collection, irrespective of the menstrual cycle phase. This could be explained by the fact that the constant OPN concentration in circulating blood may reflect the innate ability of various tissues throughout the body to produce OPN. In other words, individuals with high blood OPN levels also have a higher ability to produce OPN from follicular tissue in response to the gonadotropin surge. OPN is known to bind with several different integrin receptors, including αvβ1, αvβ3, αvβ5, α4β1, and α9β1, and CD44 antigen, also known as a hyaluronic receptor. OPN signaling via either of those receptors expressed on the surface of granulosa cells will mediate the enhancement of VEGF production in an autocrine manner. Further studies on the transcripts or proteins in early-luteinized granulosa cells accompanying the oocyte collected during IVF are required.

Luteinization is a dynamic event characterized by marked angiogenesis by VEGF produced from follicles^13^. Remarkable ascites, characteristic of OHSS, is mainly associated with the overproduction of VEGF from the ovaries, thus promoting vascular permeability^14^. It has been reported that total VEGF production from the ovaries can be estimated by multiplying the amount of follicular fluid collected during oocyte collection with its VEGF concentration^12^. AMH, LH/FSH, and E_2_ are known predictors for OHSS in the ovarian stimulation cycle; however, none of these correlated with the VEGF concentration in follicular fluid. This could be because these parameters reflect the number of mature follicles on oocyte retrieval that is proportional to the total volume of follicular fluid, on which total VEGF production depends. On the other hand, the plasma OPN level showed a positive correlation with the follicular fluid VEGF concentration during the periovulatory period regardless of the blood collection period. This finding suggests that the plasma OPN level may be a novel OHSS predictive marker independent of the conventional serum marker associated with the number of the developed follicles. Evaluation of plasma OPN along with serum AMH might be useful to identify cases with a high risk of OHSS with higher accuracy and contribute to optimizing ovarian stimulation protocols. In this study, we used the mild stimulation cycle with clomiphene citrate, and no cases of OHSS were included. Future studies should evaluate whether OPN is actually involved in the pathological condition of OHSS via a prospective observational study using the stronger ovarian stimulation cycles. A limitation of the present study was the small sample size. Hence, further investigations on more patients are necessary to confirm interpretations of our preliminary findings.

In conclusion, the blood OPN level did not significantly vary during the ovarian cycle and positively correlated with the follicular fluid OPN level irrespective of the blood-sampling period. Because plasma OPN levels also showed a positive correlation with the follicular fluid VEGF concentration at oocyte retrieval, we propose that OPN may be an independent clinical marker for evaluating the risk of OHSS.
